# Iatrogenic Upper Gastrointestinal Bleeding Following Nasogastric Tube Insertion in a Patient with Pemphigus Vulgaris

**DOI:** 10.5152/tjg.2026.25443

**Published:** 2026-01-05

**Authors:** Yusuf Bünyamin Ketenci, Asena Çakır, Gökçen Ayça Yılmaz, Ufuk Avcıoğlu, Hakan Demiröz, Mehmet Akca

**Affiliations:** 1 Department of Gastroenterology, Ondokuz Mayıs University, Samsun, Türkiye; 2 Department of Dermatology, Ondokuz Mayıs University, Samsun, Türkiye; 3 Department of Internal Medicine, Ondokuz Mayıs University, Samsun, Türkiye

Dear Editor,

Pemphigus vulgaris (PV) mainly affects the oral mucosa and skin; esophageal involvement has been controversial for many years.^1^ The first site of PV is usually the oral mucosa, and erosions seen in any area of the mouth and eroded areas seen in the vermilion of the lips are quite typical. Bullae easily rupture, causing painful erosions with epithelial debris at the edges. Nikolsky’s sign (the formation of a bullae by mechanical pressure applied to the edge of the bullae, normal skin or mucosa) is frequently seen.

Previous case reports have considered this condition quite rare, as esophageal involvement was rarely reported.^2^ However, prospective studies conducted in recent years have shown that esophageal involvement is more common than previously thought, and endoscopic detection rates range from 24% to 87%.^3^^-^^5^ A rare case of upper gastrointestinal (GI) bleeding due to PV esophageal involvement is presented herein.

A 57-year-old man with a known history of hypertension, chronic renal failure, and cerebrovascular disease was admitted to the hospital due to oliguria and decreased oral intake during home monitoring. He was admitted to the intensive care unit with a preliminary diagnosis of prerenal acute renal failure based on chronic renal failure. Bloody content was observed coming from the mouth and the nasogastric (NG) tube inserted with a feeding plan. Then, melena occurred. Before the NG tube, there were no bleeding symptoms or findings like hematemesis or melena.

An esophagogastroduodenoscopy revealed giant black-red circumferential hematomas, especially in the distal esophagus. There were no pathological findings in the computerized tomography. There was no response to the high-dose proton pump inhibitor (PPI), antacid, and sucralfate treatment, and the appearance was still similar in the control endoscopy (Figure 1a-c). Because of the mucosal fragility, we could not take any biopsy from the esophageal lesions.

In the detailed history scan, it was seen that the patient was diagnosed with cutaneous PV 1 year ago but remained untreated.

After the patient’s history was recognized, the patient’s physical examination revealed loose bullae with clear and hemorrhagic content that rapidly transformed into post-bullous erosions on a non-erythematous base, consistent with PV, which had been ongoing for the last 2 years. There was no itching in the lesions, and the Nikolsky sign was positive. Eroded areas were also seen on the patient’s buccal mucosa. During the patient’s follow-up, because of a reduction in the hemoglobin levels from 9.5 mg/dL to 5.7 mg/dL in the complete blood count, he required 6 units of erythrocyte suspensions.

Significant improvement was observed in 1 week with methylprednisolone treatment at 0.5 mg/kg. After that, hemoglobin levels were stable. And melena disappeared.

The PPI treatment was added for bleeding prophylaxis. The patient was monitored without oral feeding until clinical improvement was observed.

After 10 days with glucocorticoid treatment, in the control esophagogastroduodenoscopy, the esophagus was completely normal. There were not any signs of hematomas. This situation supports that the hematomas were Nikolsky sign-positive mucosal bullae. Informed written consent was obtained from the patient.

The most common symptoms in PV patients with esophageal involvement are dysphagia (57.1%) and odynophagia (21.4%), and some patients may also be asymptomatic.^5^ Studies have reported that esophageal symptoms are ignored in many patients without endoscopic examination. However, in a study by Rao et al,^3^ esophageal involvement was detected endoscopically in 67.5% of PV patients, even if they did not have symptoms. This shows that the effect of PV on the esophagus is not limited to clinical symptoms and that detailed endoscopic examinations may be important.

Okamura et al^4^ applied the endoscopic Nikolsky test to evaluate the fragility of the esophageal mucosa and obtained a positive result of 87%, even in normal-appearing mucosa. This finding provides a new perspective on understanding the subclinical involvement of PV in the esophagus. However, Nikolsky test positivity has not yet been accepted as a specific criterion for esophageal involvement, and further studies are required.

In this case, the patient had significant hematemesis after NG tube placement, and extensive mucosal damage was observed in the endoscopic examination. In this situation, the NG tube caused a positive Nikolsky injury in the esophagus. So, iatrogenic GI bleeding occurred.

As in our case, presentation with life-threatening bleeding of upper GI bleeding is a rare manifestation of PV esophageal involvement. To the best of our knowledge, this is the first report of iatrogenic upper GI bleeding secondary to NG tube insertion in a patient with PV. In this respect, we believe our case has scientific value. If an NG tube needs to be inserted in patients diagnosed with PV, care should be taken to avoid iatrogenic bleeding.

Although esophageal involvement is accepted to be widespread in light of recent information, it is still controversial whether endoscopy should be routinely performed in all PV patients. In the literature, although severe involvement of the esophageal mucosa was detected during the active disease period, mucosal recovery was reported to be quite high after immunosuppressive treatment. In the study conducted by Galloro et al,^5^ it was shown that mucosal recovery was achieved in all patients in control endoscopies performed after corticosteroid treatment was started. In our case, significant improvement was observed in esophageal involvement symptomatically and endoscopically after initial treatment.

In line with these findings, the following 2 basic suggestions emerge: First, endoscopy generally does not provide additional information during the active disease period because esophageal involvement is already present at a high rate. Another suggestion is that endoscopy is necessary for the presence of persistent dysphagia, odynophagia, and bleeding findings after treatment, as this may suggest a pathology other than PV (e.g., stricture, candidiasis, or gastroesophageal reflux disease).

In conclusion, large-scale prospective studies are needed on the esophageal presentation, clinical management, and long-term outcomes of PV. In particular, the development of non-invasive methods for early diagnosis may facilitate patient management.

As limitations for this case, we were unable to obtain biopsies from the esophagus. Therefore, we couldn’t perform immunochemistry and immunofluorescence. But we recognize the clinical features.

## Figures and Tables

**Figure 1.**
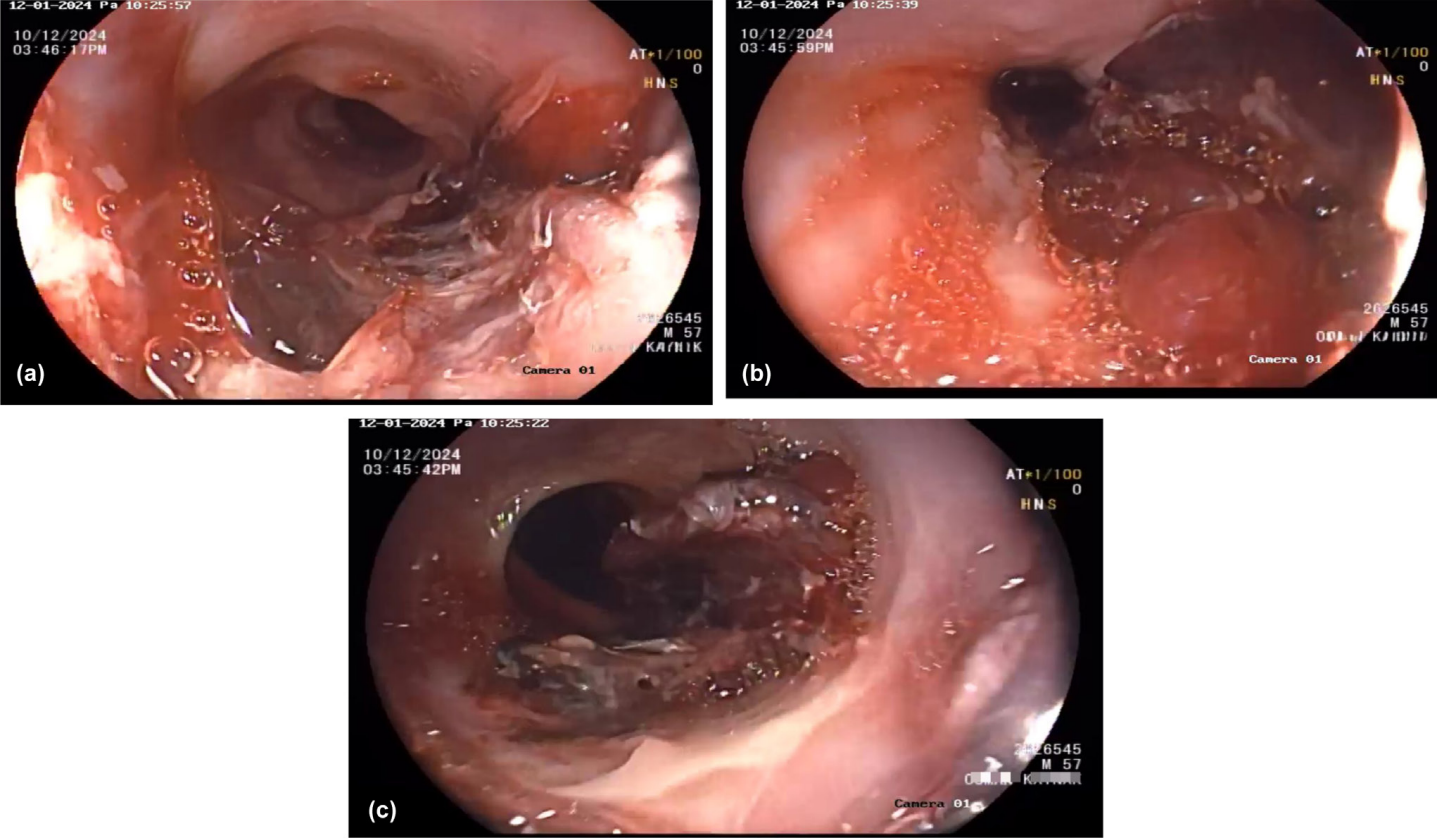
Esophageal endoscopic finding: Linear erosions with haematoma, mucosal fragility. a: proximal esophagus; b: middle esophagus; c: distal esophagus.

## Data Availability

The data that support the findings of this study are available on request from the corresponding author.
